# Clinical Features That Evoke the Concept of Disinhibition in Tourette Syndrome

**DOI:** 10.3389/fpsyt.2020.00021

**Published:** 2020-02-25

**Authors:** Lille Kurvits, Davide Martino, Christos Ganos

**Affiliations:** ^1^ Department of Neurology, Charité University Hospital, Berlin, Germany; ^2^ Department of Clinical Neurosciences, University of Calgary, Calgary, AB, Canada

**Keywords:** Tourette syndrome, tics, inhibitory control, disinhibition, Gamma aminobutyric acid, basal ganglia

## Abstract

The capacity to efficiently control motor output, by either refraining from prepotent actions or disengaging from ongoing motor behaviors, is necessary for our ability to thrive in a stimulus-rich and socially complex environment. Failure to engage in successful inhibitory motor control could lead to aberrant behaviors typified by an excess of motor performance. In tic disorders and Tourette syndrome (TS) — the most common tic disorder encountered in clinics — surplus motor output is rarely the only relevant clinical sign. A range of abnormal behaviors is often encountered which are historically viewed as “disinhibition phenomena”. Here, we present the different clinical features of TS from distinct categorical domains (motor, sensory, complex behavioral) that evoke the concept of disinhibition and discuss their associations. We also present evidence for their consideration as phenomena of inhibitory dysfunction and provide an overview of studies on TS pathophysiology which support this view. We then critically dissect the concept of disinhibition in TS and illuminate other salient aspects, which should be considered in a unitary pathophysiological approach. We briefly touch upon the dangers of oversimplification and emphasize the necessity of conceptual diversity in the scientific exploration of TS, from disinhibition and beyond.

## Inhibitory Control in Health and Disorder

The capacity to efficiently control motor output in order to fulfill a desired outcome is a fundamental characteristic of our behavior. Beyond the selection and initiation of context-specific behaviors (e.g., waving the hand to signal familiarity), the ability to either refrain from executing prepotent actions (e.g., not slamming a door) or to timely disengage from an ongoing motor behavior (e.g., stop speaking at the beginning of an opera) is necessary for our capacity to thrive in a stimulus-rich and socially complex environment. The importance of the inhibitory qualities of our behavior is further emphasized by religious, moral, social, and legal regulatory codes, which typically penalize lack of self-control, often manifesting as non-conforming or inappropriate behaviors. Importantly, effective self-regulatory control constitutes a potential neurodevelopmental marker of well-being, as people who were able to better control their actions early in life were found to have improved psychosocial functioning, including resilience and coping with stressors, sense of self-worth and higher degree of education as adults (1).

A particular, and perhaps the most basic aspect in the behavioral science of self-regulatory control (or simply “self-control”) (2), is the study of motor responses. However, the scientific exploration of motor inhibition is notoriously difficult, as successful control over movement implies a behavior that never occurs (3). Therefore, psychology often examines the effects of insufficient behavioral inhibition in certain neuropsychiatric and movement disorders typified by an excess of behavioral motor output (4). On the one hand, these disorders exemplify the importance of efficient inhibitory control over motor output. On the other hand, they also provide the unique opportunity to scientifically examine the neural circuitry of motor inhibition. Indeed, in hyperkinetic movement disorders, loss of inhibition at different neuronal levels and networks has been associated with the manifestation of different abnormal motor behaviors (4). For example, loss of inhibition in spatial and temporal sensorimotor processing is a common pathophysiological finding in some forms of isolated dystonia (5–7) and loss of cerebellar inhibitory control over the motor cortex is suggested to be an underlying factor driving the manifestation of cortical myoclonus (8, 9)

Perhaps the most popular example of inhibitory dysfunction in medical literature and movement disorders are tic disorders, and particularly Tourette syndrome (TS), the most common tic disorder encountered in clinics. TS is defined by the presence of at least two motor tic behaviors and one vocal tic behavior for a minimum period of a year, manifesting before the age of 18 (10). Although there are several clinical features of TS that evoke the concept of disinhibition, scientific studies assessing different inhibitory functional domains have provided mixed results (11–14). Thus, the unitary concept of TS as a disorder of inhibitory control remains controversial (15–18). In order to provide some clarity to this critical issue, we here first present a list of clinical features which evoke the concept of disinhibition classified in distinct categorical domains. We then provide evidence and an overview of studies on TS pathophysiology which support the view of inhibitory dysfunction. We subsequently critically dissect the concept of disinhibition in TS and highlight additional aspects that should be considered in TS pathophysiology. Finally, we discuss the dangers of oversimplification and emphasize the necessity of conceptual diversity in the scientific exploration of TS. This should incorporate additional evidence-driven approaches, including pathophysiological frameworks such as enhanced reinforcement learning, impaired predictive coding for action control, and abnormal body-focused metacognitions.

## The Motor Manifestations of TS That Evoke the Concept of Disinhibition

### Simple Tics – Uncontrollable Surplus Fragments of Behavior

Tics are the prototypical and defining manifestation in TS and indeed the main clinical feature evoking the notion of disinhibition. Historically, there has been some confusion as to the exact classification of tic behaviors and their distinction from other pathological conditions of excessive motor output, as spasms or jerks (19). Moreover, there has been a long dispute, — which in some regard is still ongoing (20)— as to whether tics can be truly distinguished from voluntary actions. Following Meige and Feindel's scholarly treatise on “Les tics et leur traitement”, a first definition of tics was provided, and surprisingly little has changed since then. Tics are movements or sounds that resemble physiological actions, but appear repetitive and are inopportune to contextual cues from the environment (21, 22). Accordingly, tics represent excessive motor behaviors that are superimposed to ongoing voluntary motor output, but are inflexible and often appear exaggerated in intensity and frequency (23). There is marked intra- and interpersonal variability of tic behaviors; any possible movement or sound can constitute a tic. However, there are specific features, particularly at the onset of tic behaviors, which are characteristic for TS. First, individual tic behaviors are typically brief, occur suddenly and involve only a limited number of muscles (also termed simple tics), often resembling fragments of normal voluntary motor behavior. Second, many tics involve the face. Third, tics are amenable to a specific cognitive process of effortful voluntary inhibitory control and can thereby be typically suppressed on demand (17). These specific characteristics of simple tic behaviors, particularly their surplus and exaggerated nature superimposed to ongoing voluntary actions, as well as their brief, sudden and socially inopportune character (e.g., facial tics appear as conspicuous behaviors in the absence of conveyed social meaning), and their susceptibility to voluntary control evoke the concept of motor disinhibition.

### Complex Tics – Seemingly Purposeless But Socially Disruptive Actions

Beyond simple tics, patients with TS may also often exhibit complex tic behaviors. These resemble goal-directed actions (e.g., shrugging shoulders, waving hello, jumping on the spot, uttering words or short sentences etc.) but are repetitive and without apparent purpose. Although the prevalence and clinical characteristics of the entire range of complex tics compared to simple tics remains underdetermined, certain behaviors, to include echo-, pali-, and coprophenomena have been better studied. Among these and perhaps owing to their vexing and socially disruptive nature, coprophenomena received more clinical attention, and are typically viewed as the pivotal example of behavioral disinhibition in TS.

Coprophenomena are defined as the unintended expression of socially obscene actions (copropraxia) and/or utterances (coprolalia) (24). Despite their notoriety, they only occur in less than 30% of TS patients (24). Coprophenomena typically first appear around the age of 11 years (24), indeed with a lag of several years after overall tic onset. Coprolalia is more prevalent than copropraxia, and most patients who exhibit copropraxia also have coprolalia (24). No sex differences were found in the expression of coprophenomena in TS, but there were strong associations with tic severity, as well as the overall number of neuropsychiatric comorbidities such as OCD, self-injurious behavior (SIB, also see below), and anger control issues (24, 25). Moreover, a link was established between coprophenomena and non-obscene socially inappropriate type of behaviors, as well as sexually inappropriate behaviors (also see below). The prevalence of these types of behaviors in patients who exhibit coprophenomena and are severely affected by tics lend support to the notion of a broader inhibitory deficit, which afflicts both patterns of movement, and also overall expressions of behavior.

**Figure 1:**
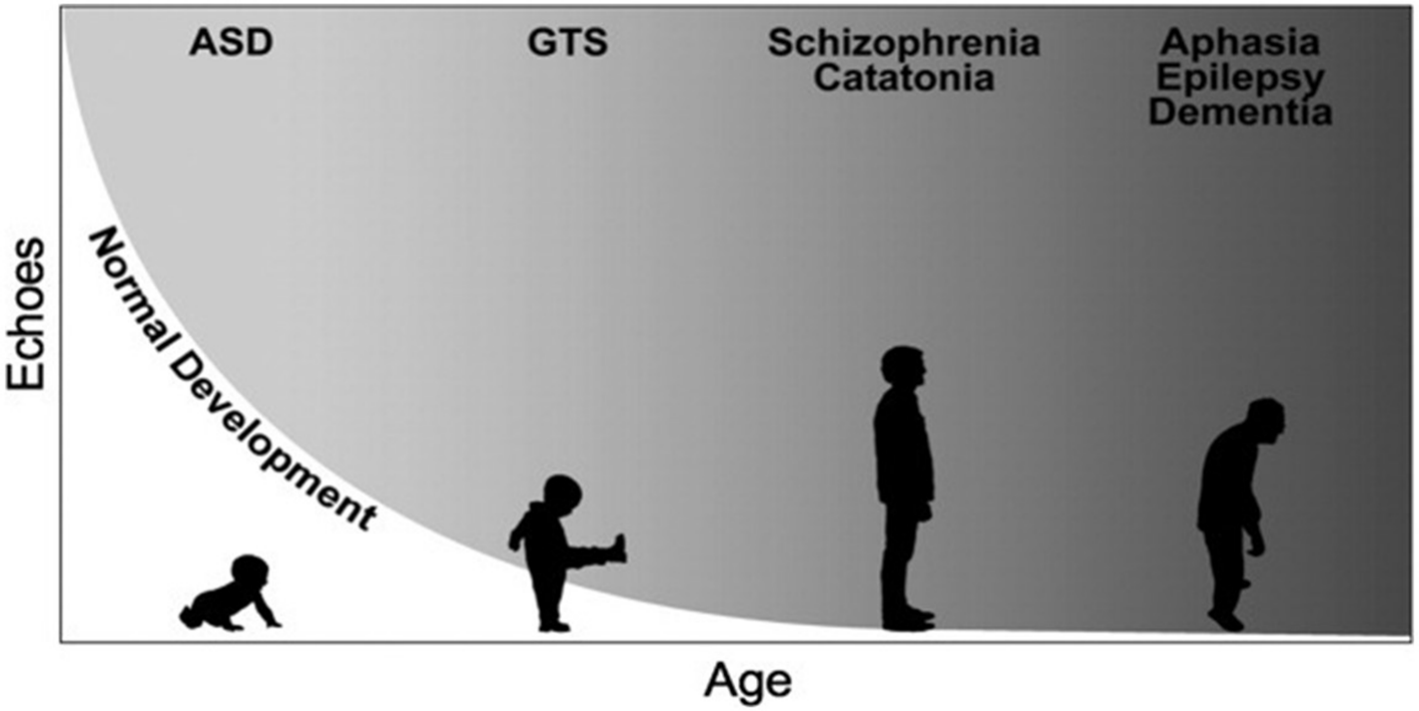
Simplified representation of the developmental trajectories of echophenomena in health and disease. Echophenomena are present in normal childhood development, with a gradual reduction throughout the first three years of life (depicted trajectory in white). Gray shades demonstrate the persistence or reemergence of echophenomena as a sign of underlying neuropsychiatric disorders, e.g., in autism spectrum disorders (ASD), Gilles de la Tourette syndrome (GTS) or others [figure republished with permission from Wiley publishing, (26)].

Echophenomena denote the imitation of actions (echopraxia) or sounds (echolalia), without the prerequisite of explicit awareness over their occurrence (26). Although in TS echophenomena fall within the rubric of complex tic behaviors, they are encountered in many other neuropsychiatric disorders, also in the overt absence of tics (**Figure 1**). Thereby, it remains unclear whether the pathophysiological underpinnings of echophenomena are similar to that of other tic behaviors. Indeed, echophenomena are characterized by their immediacy with the external environment, as they are directly generated by it as externally triggered behaviors. Despite methodological differences between studies (e.g., direct observation using standardized protocols vs clinical examination vs self-report), echophenomena appear to be present in about a third of patients with TS and similar to coprophenomena, echolalia is noted more commonly than echopraxia (25). One study provided experimental evidence on the exact characteristics of echopraxia and showed that patients most commonly echoed behaviors, which belonged to their tic repertoire (27). Compared to patients who do not report echophenomena, the presence of echo- behaviors was positively associated with tic severity and the overall burden of neuropsychiatric comorbidities (25). Given the transient neurodevelopmental nature of echophenomena in healthy development, and their persistence or reemergence later in life in TS, as well as the overall burden of behavioral difficulties in these patients, a putative inhibitory deficit over the control of pre-wired patterns of socially-triggered motor behaviors has been suggested (26).

Paliphenomena refer to the repetition of self-generated movements (palipraxia) or sounds/words (paliphonia/palilalia). Similar to echophenomena, paliphenomena may present in a whole range of neuropsychiatric conditions, particularly in disorders, such as those affecting the frontal lobes, where loss of behavioral control and social disinhibition are characteristic (28, 29). However, not all cases with brief repetitive utterances (e.g., the repetition of phonemes or syllables) are palilalic behaviors, and indeed a distinction from stuttering should be considered. In TS paliphenomena are often induced through an echo-behavior. For example, saying “hello” to a patient may result in the repetitive utterance of several “hellos” in return. Although it is unclear why patients need to repeat certain phonemes or actions, the sheer phenomenology of paliphenomena resembles the observable inflexible behavioral output of patients with OCD, where compulsions such as repetitive checking, washing, or touching are characteristic. Therefore, studies have sought to explore similarities and differences between the two phenomena, as indeed paliphenomena may also occur in TS in the absence of overt obsessive-compulsive behaviors (30, 31). Compared to patients with OCD, TS-only patients tend to display more egosyntonic automatic repetitive behaviors like mental play (e.g., repetitive, often intended as pastime and mostly not unpleasant impulses or cognitions, including images or sounds) (32) touching and just-right phenomena (e.g., the need to perform a tic or a compulsion until it is experienced “just right”) (33). This is in contrast to the distressing, egodystonic and occasionally aggressive repetitive thoughts, contamination worries, and washing behaviors of OCD (31). There is a distinction within the spectrum of repetitive behavior in TS (30). On one side are some of the repetitive behaviors, which are goal-directed and volitional to reduce anxiety, such as the OCD-like checking, washing hands, and on the other side are tic-like repetitive behaviors, which are more egosyntonic, automatic, sometimes even referred as “impulsions”, which belong to paliphenomena (30–32). As in the previous two types of complex tic behaviors (copro- and echophenomena), patients with more severe tics and neuropsychiatric comorbidities will often exhibit paliphenomena, particularly palilalia (25). The repetitive, excessive, inflexible and purposeless nature of paliphenomena has, therefore, also contributed to the view that in TS inhibitory deficits are related with an inability to properly measure and dosage behavioral output.

## The Sensory Manifestations of TS That Evoke the Concept of Disinhibition

### Premonitory Urges – Pathological Interoceptive Experiences in Excess

Beyond the motor manifestations of simple and complex tic behaviors evoking the concept of disinhibition described above, patients with TS also report sensory abnormalities, which may equally serve as markers of inhibitory dysfunction. Firstly, the majority of adolescents and adults with tics describe sensations preceding their tic behaviors, which are commonly known as premonitory urges. Although descriptions of these excessive bodily experiences often lack precision, patients report a range of perceived phenomena, spanning from a somatic urge to move, to increased tension, an urge to apply pressure or stretch particular body parts, an ache, itch, tingle, and others (34). Most importantly, premonitory urges are often viewed as the defining pathophysiological element, which drives and/or perpetuates tic behaviors (35). Indeed, most patients, particularly in adolescence and beyond, report premonitory urges preceding their tics (25, 36) and often view their tic behaviors as a voluntary response to the unpleasant sensory experience. Despite the apparent straightforward relation between premonitory urges and tics (i.e., tics are the result of premonitory urges), the exact pathophysiological relevance of these phenomena remains unclear (37, 38), and several models have been proposed to explain the presence of excessive bodily sensations (35, 36, 39–41). According to one promising line of research, the capacity to perceive premonitory urges is related to the overall capacity to perceive interoceptive signals (41, 42), possibly providing an explanatory framework to understand the associations of premonitory urges with other clinical symptoms, such as anxiety or obsessive-compulsive behaviors (25, 43–46). Interestingly, patients with TS are less well able to perceive their own physiological interoceptive signals (41, 42, 47).

### Sensory Hypersensitivity – Perceived Exteroceptive Surplus

Heightened sensitivity to multimodal exteroceptive stimuli is another salient clinical feature of TS that evokes the concept of disinhibition. For example, increased perception of tactile, auditory and visual stimuli was noted in adolescents and adults with TS (48–50). Crucially, one of the studies identified four perceptual domains which discriminated TS subjects from healthy controls. These domains included perceived stimulus intensity, distractibility, stimulus discrimination and the capacity to attend to sensory stimuli during phases of stress/fatigue (49). Given neurophysiological evidence of deficient gating of sensory input in studies of prepulse inhibition in TS (reduced inhibition of response to a single stimulus, e.g. startle-response following a prepulse stimulus) (49, 51–54), a framework of sensorimotor disinhibition at the basis of tic disorders was further supported. However, the only three studies that clinically examined sensory thresholds in adolescents and adults with TS did not identify significant differences from healthy controls, despite patient reports of increased somatic experiences (55–57).

## Other Behavioral Manifestations of TS That Evoke the Concept of Disinhibition

### Impulsivity and Attention-Deficit Hyperactivity Disorder

Already in 1902 Meige and Feindel commented on the role of impulsivity as a distinctive feature of people with tics and TS. Their observation has since been confirmed in numerous clinical studies (58, 59). Together with inattention and hyperactivity – the major clinical subtypes of attention deficit hyperactivity disorders (ADHD) – the lifetime prevalence of these behaviors exceeds 50% (59). Beyond the typical neurocognitive difficulties due to ADHD affecting executive functions (e.g. response inhibition), overall academic performance and psychosocial functioning (60), patients with tics and ADHD more often exhibit disruptive behaviors like those related to oppositional defiant disorder (ODD) and conduct disorder (61) and episodic rage outbursts, also known as rage attacks, compared to patients with tics without ADHD. This latter set of behaviors is characterized by discrete episodes of failure to resist aggressive impulses leading to overt behaviors, where the degree of aggression is grossly disproportionate to any precipitating stressors (62). A body of evidence supports the association between these different behavioral manifestations and ADHD comorbidity in patients with tic disorders. For example the presence of explosive outbursts in patients with tics has been repeatedly documented in observational clinical studies that reported how the vast majority of patients with rage attacks had more than one comorbid disorder, specifically ADHD or OCD, alongside TS (63–65). Apart from being more prevalent in TS children and adolescents with co-existing ADHD, ODD, and conduct disorder display a greater stability of symptom severity in this patient subgroup compared to TS patients without ADHD, indicating that the development of ODD and conduct disorder in TS is influenced by ADHD comorbidity (66). Another study reported a differential impact on ODD symptom domains exerted by ADHD (associated with headstrong or defiant domain of ODD) and obsessive-compulsive behaviors (associated with the irritability domain of ODD) (67).

### Non-Obscene and Other Socially Inappropriate Behaviors

Another putative aspect of behavioral disinhibition in TS are non-obscene inappropriate behaviors (68). These sets of behaviors are characterized by the impulse to make insulting or inappropriate comments or actions related to an individual or a situation. They differ from coprophenomena in two main aspects. First, coprophenomena are typically purposeless, unintended behaviors, whereas non-obscene socially inappropriate behaviors are externally triggered and stimulus-bound. Second, the content of these behaviors does not involve swearing or obscene derogatory actions. However, non-obscene socially inappropriate behaviors and coprophenomena may often manifest together (68, 69). A carefully conducted study by Eddy and Cavanna (69) in 60 adults with TS found that that presence of non-obscene socially inappropriate behaviors was associated with obsessive-compulsive behaviors, ADHD, mental coprolalia, and poor quality of life.

A brief note should also be made to inappropriate sexual behaviors in TS, which were indeed commonly described in older literature (70–72). Different from non-obscene socially inappropriate behaviors, reported inappropriate sexual behaviors range from inappropriate talks and jokes about sex, over to exhibitionism and paraphilic behaviors. Although this area of research remains underexplored, these behaviors were shown to be more prevalent in samples of TS patients compared to controls, particularly in the presence of comorbid ADHD and coprophenomena (70, 73).

### Self-Injurious Behaviors (SIB)

The umbrella term of SIBs encompasses different behaviors leading to bodily self-harm that are neither characteristic for patients with TS nor uniform in their clinical presentation or underlying pathophysiology. However, patients with tics and TS often exhibit SIBs, with an overall prevalence estimated between 15% (73) and 39% (25). SIBs in TS include repetitive skin picking or scratching, head-banging, head or body punching/slapping, body-to-hard-object banging, and poking sharp objects into body parts (74). In a carefully conducted study by Mathews and colleagues, 29% of 297 individuals with TS showed some form of SIB. Importantly, the severity of SIB allowed discriminating between clinical determinants of self-harm, further corroborating pathophysiological diversity. In milder forms of SIBs there was a correlation with obsessive-compulsive symptoms, including violent and aggressive obsessions/compulsion. Impaired impulse control (rage and risk-taking behaviors) and tic severity were associated with severe SIB (75), in line again with the notion of an extensive inhibitory deficit governing both inappropriate and self-harming behavioral output, as well as the overall expression of tics.

## Disinhibition as a Pathophysiological Framework in TS: Sides of a Controversy

### A Congruent View of Disinhibition as the Core Abnormality of Tics and TS

**Figure 2:**
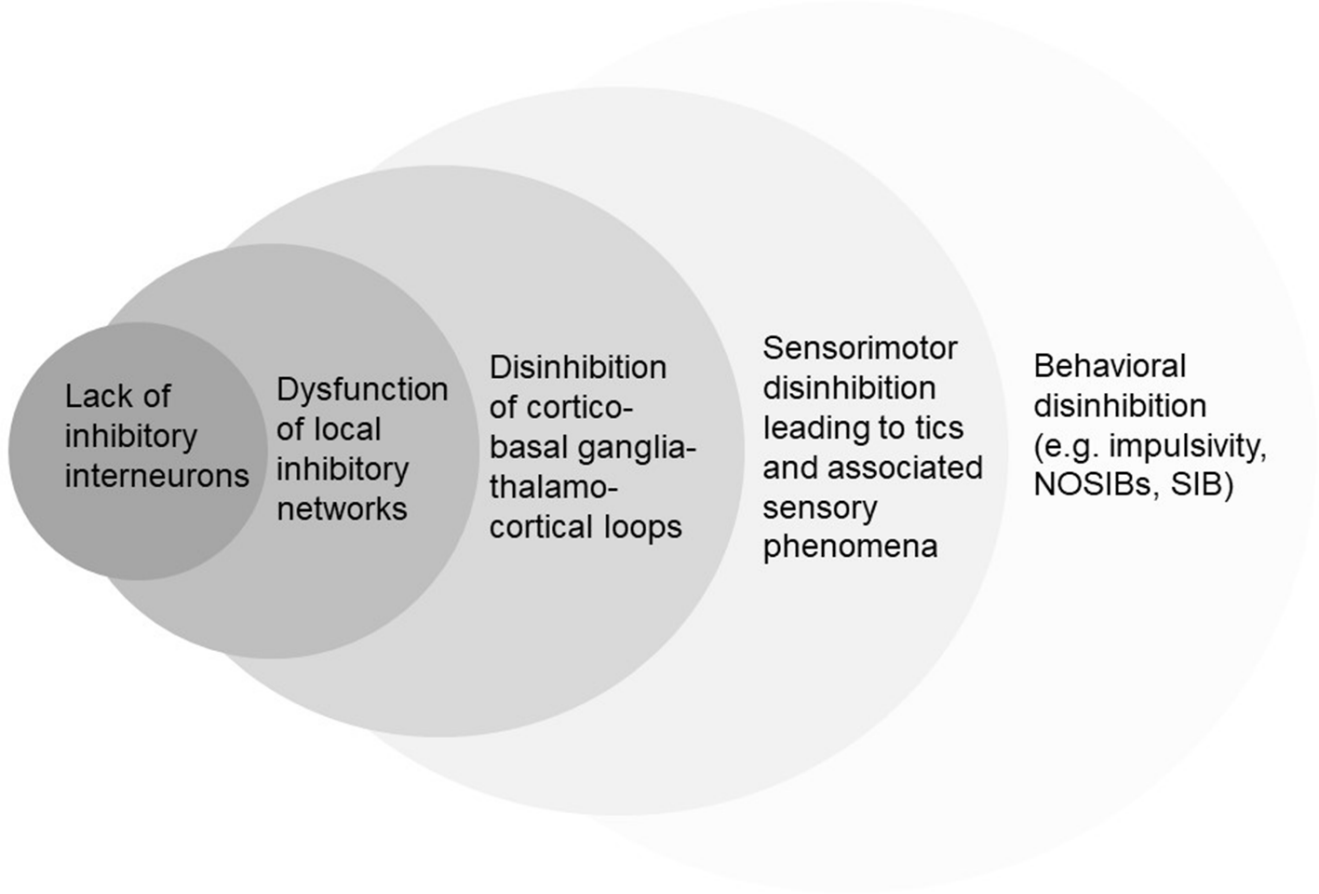
Simplified hierarchic representation of different conceptual levels of disinhibition in TS. NOSIBs; Non-Obscene Socially Inappropriate Behaviors. SIB; Self-Injurious Behaviors.

A pathophysiological framework of disinhibition could explain the illustrated motor, sensory, and other behavioral characteristics of TS (**Figure 2**). Interestingly, this view has a strong historical background. Tics and their associations were considered to afflict those with “weak control over their actions” (21), with the extent of clinical abnormalities suggested to correlate to the severity of the underlying inhibitory dysfunction. Indeed, after a century of clinical research, experimental data provided strong support for the disinhibition theory (76, 77).

From a neuroanatomical perspective, two neuropathological studies demonstrated a reduction of inhibitory gamma-aminobutyric acid-ergic (GABA) and cholinergic interneurons in the striatum of patients with TS (78, 79). The abnormalities of the cholinergic interneuronal population were specifically found in the associative and sensorimotor parts of the striatum, in areas that were previously predicted to be involved in the pathophysiology of abnormal tic behaviors (80). The functional relevance of these findings was further supported by animal models of tic disorders. Indeed, pharmacological striatal GABAergic disinhibition has been associated with motor and behavioral abnormalities that may well fall within the tic disorder spectrum (81–83).

Cortical neurophysiology has also provided evidence of inhibitory dysfunction in TS. Transcranial magnetic stimulation measures of both short-interval intracortical inhibition (SICI) and short-afferent inhibition (SAI) were found reduced in TS (84–89). Also, using magnetoencephalographic recordings during the execution of brief voluntary actions, an imbalance between local inhibitory-excitatory neuronal populations in motor and sensory cortical circuits has been previously demonstrated (90, 91). In addition, and even though not specific to TS, prepulse inhibition of the startle response was found deficient in people with tic disorders (49, 51–53). Finally, both behavioral and heritability studies also supported a notion of a pervasive disinhibition trait. A recent meta-analysis on the behavioral performance of patients with TS compared to controls in different inhibitory tasks revealed a mild but significant excess of inhibitory deficits in patients (15) compared to control subjects. The difference was more pronounced for tasks exploring verbal rather than pure motor inhibitory measures, and showed a positive correlation between the degree of these deficits and tic severity. An examination of a large data-set of 1,191 people with TS and 2,303 of their first-degree relatives concluded that behaviors such as copropraxia, palilalia, and others (also noted above; termed in study as “socially disinhibited behaviors”) represent a heritable TS subphenotype, associated with more neuropsychiatric comorbidities (e.g., ADHD, OCD), earlier tic onset and overall higher tic severity (92).

### Challenging the View of Disinhibition as the Core Abnormality of Tics and TS

Despite the wealth of presented data, not all efforts to establish disinhibition as an overarching pathophysiological theme in TS have been successful. Importantly, a critical dissection of some of the aforementioned studies provides more complex insights into the underlying circumstances that lead to tics and their associations. First, the aforementioned neuropathological evidence stems from brain samples of a small cohort of five patients. These patients were on average 18 years younger compared to healthy controls (79) and had been previously chronically exposed to psychotropic medications, including antipsychotics. Second, although the pharmacological striatal GABAergic disinhibition model for tics has provided invaluable information to the putative pathophysiological mechanisms of the syndrome (93), it is important to note that it remains unclear whether the observed animal behaviors following disinhibition are indeed tics. In fact earlier studies of similar pharmacologic models referred to the generated abnormal motor output as myoclonus or dyskinesias (94–96), which highlights the difficulty of using a consistent phenomenological labeling for these toxin-induced motor behaviors. Most importantly, not only GABAergic but also other neurotransmitter animal models (97, 98) have been proposed to explain clinical findings, and thereby contribute to the pathophysiology of the syndrome. Overall, it may be argued that the inability to examine whether some of the abnormal motor output is preceded by premonitory urges, or whether it is actively suppressible question the validity of all animal models of tic behaviors. Third, the neurophysiological study of tic disorders has not only provided evidence suggestive of disinhibition, but has also revealed normal or supra-normal inhibitory functions. Some general measures of motor cortical excitability, e.g. resting and active motor thresholds, are not different in TS patients from those in healthy controls (84), whereas other measures such as the recruitment of motor-evoked potentials (input/output curves) suggest reduced baseline excitability (84, 88, 99). Similarly, some magnetoencephalographic cortical event-related studies also revealed patterns of enhanced inhibitory function (100, 101). Although these findings are often suggested to reflect active symptom compensation (102), they do highlight that the functionality of relevant inhibitory networks in TS is intact and that these can be recruited when necessary. This view is also supported by a wealth of neuroimaging studies, the majority of which examined cross-sectional samples, where it is often difficult to distinguish whether findings (e.g., prefrontal cortical volumes or task-related activations) point towards deficient inhibition, enhanced compensation, or both (16, 76). A crucial factor in this regard is also the age of the studied population. The inhibitory capacity of children and adolescents with tic disorders and TS could well differ from adults and indeed drawing firm conclusions from comparing data between cross-sectional samples of patients is strongly hampered by this limitation. Finally, although an overall mild effect was found with regard to behavioral evidence of disinhibition of action, the significant heterogeneity of the examined studies does not allow drawing firm conclusions (15). The conceptual fallacy of a framework where tics represent fragments of disinhibited actions was previously discussed (17).

## Why Is Investigating Disinhibition in TS Important? Conclusive Remarks

The ongoing discussion on whether motor and behavioral disinhibition is a pivotal mechanism in tic disorders should not remain a mere academic exercise but should be channeled towards a more granular description of the clinical spectrum of TS and other chronic tic disorders. There is little doubt that the debate is fueled by evidence partly supporting and partly refuting a unitary conceptualization of tics and related repetitive behaviors as the result of insufficient capacity to ‘hold in' unwanted actions. Moreover, recent work within the field of cognitive neuropsychology proposed alternative conceptualizations of tic genesis, among which are the formation of pathologically reinforced stimulus-response associations or sensorimotor programs (103–105), the generation of physiological motor commands to match abnormal sensorimotor priors (106), or the release of motor output that results from excessive body-focused metacognitions (107). None of these alternative conceptualizations is either based on defective inhibition, or categorically excludes the contribution of defective inhibition to the genesis of tics. For example, modulation of striatal GABAergic interneurons was demonstrated to mediate reinforcement-related cholinergic striatal interneuronal function (108). This could explain the presence of both quantitative abnormalities in GABAergic interneuronal populations in neuropathological studies in people with TS (78, 79) and the existence of enhanced reinforcement learning (103, 105). A system with these critical alterations in its basic sensorimotor circuitry may also be fraught in the relative weighing of information processing and monitoring (109). In turn this could lead to the presence of abnormal metacognitions for sensorimotor events (107, 110), including changes in the experience of interoceptive signals (41) and voluntary actions (111, 112).

The limited evidence of numerically defective or dysfunctional inhibitory interneurons in the brain of patients with TS, as well as the legitimate doubt that the striatal disinhibition animal model of tics yields sufficient face validity vis-à-vis human disease, indicate that preclinical or *post mortem* studies did not provide a solution to the debate in object. In the wake of neuropathological research yielding more generalizable results and animal models demonstrating greater validity for tic disorders in humans, clinical research should invest more in the exploration of biological markers or endophenotypes related to disinhibition, and evaluate how these correlate with specific phenotypic subtypes of the TS spectrum. Importantly, clinical research has already shown that the comorbidity profile should be incorporated in the definition of these phenotypic subtypes, with particular attention to ADHD. Neurophysiological markers of defective inhibition, e.g. those based on TMS-EMG and pre-pulse inhibition paradigms, should be assessed in association with specific clinical characteristics of the syndrome, such as presence/absence of echo-, pali-, coprophenomena, socially inappropriate behaviors, or self-injurious behaviors. Conversely, a similar clinic-physiological correlation should be performed for those markers (e.g., event-related potentials) that yielded evidence of preserved or even enhanced inhibition. Moreover, it would be interesting to assess if this enhanced inhibition truly correlates with a performance gain in specific executive tasks for which compensation by increased cognitive control has been hypothesized in this condition.

With this approach in mind, the puzzling dilemma of disinhibition in TS would be re-converted into a multidisciplinary experimental factory that could lead to the identification of specific clinical and pathophysiological subtypes differing on the basis of decreased or increased motor and behavioral inhibitory capacity. Such a novel course of phenotype subgrouping within the TS spectrum would bear important implications for the personalization and the selection of appropriate behavioral interventions for tics, and for the identification of biomarkers that would help monitoring treatment efficacy and forecasting the evolution of tic behaviors when patients transition from neurodevelopmental stages to maturity.

## Author Contributions

LK drafted the manuscript. DM revised the manuscript. CG made substantial contributions to the conception of the work and revised the manuscript.

## Funding 

This research project was supported by a grant from the VolkswagenStiftung (Freigeist) held by CG.

## Conflict of Interest

The authors declare that the research was conducted in the absence of any commercial or financial relationships that could be construed as a potential conflict of interest.
